# Root and shoot competition lead to contrasting competitive outcomes under water stress: A systematic review and meta-analysis

**DOI:** 10.1371/journal.pone.0220674

**Published:** 2019-12-11

**Authors:** Alicia J. Foxx, Florian Fort

**Affiliations:** 1 Plant Biology and Conservation; Northwestern University, Evanston, Illinois, United States of America; 2 Plant Science and Conservation, The Chicago Botanic Garden, Glencoe, Illinois, United States of America; 3 CEFE, Montpellier SupAgro, Université de Montpellier, CNRS, EPHE, IRD, Université Paul Valéry, Montpellier, France; Irstea, FRANCE

## Abstract

**Background:**

Competition is a critical process that shapes plant communities and interacts with environmental constraints. There are surprising knowledge gaps related to mechanisms that belie competitive processes, though important to natural communities and agricultural systems: the contribution of different plant parts on competitive outcomes and the effect of environmental constraints on these outcomes.

**Objective:**

Studies that partition competition into root-only and shoot-only interactions assess whether plant parts impose different competitive intensities using physical partitions and serve as an important way to fill knowledge gaps. Given predicted drought escalation due to climate change, we focused a systematic review–including a meta-analysis on the effects of water supply and competitive outcomes.

**Methods:**

We searched ISI Web of Science for peer-reviewed studies and found 2042 results. From which eleven suitable studies, five of which had extractable information of 80 effect sizes on 10 species to test these effects. We used a meta-analysis to compare the log response ratios (lnRR) on biomass for responses to competition between roots, shoots, and full plants at two water levels.

**Results:**

Water availability treatment and competition treatment (root-only, shoot-only, and full plant competition) significantly interacted to affect plant growth responses (p < 0.0001). Root-only and full plant competition are more intense in low water availability (-1.2 and -0.9 mean lnRR, respectively) conditions than shoot-only competition (-0.2 mean lnRR). However, shoot-only competition in high water availability was the most intense (— 0.78 mean lnRR) compared to root-only and full competition (-0.5 and 0.61 mean lnRR, respectively) showing the opposite pattern to low water availability. These results also show that the intensity of full competition is similar to root-only competition and that low water availability intensifies root competition while weakening shoot competition.

**Conclusions:**

The outcome that competition is most intense between roots at low water availability emphasizes the importance of root competition and these patterns of competition may shift in a changing climate, creating further urgency for further studies to fil knowledge gaps addressing issues of drought on plant interactions and communities.

## Introduction

**Fig 1:**
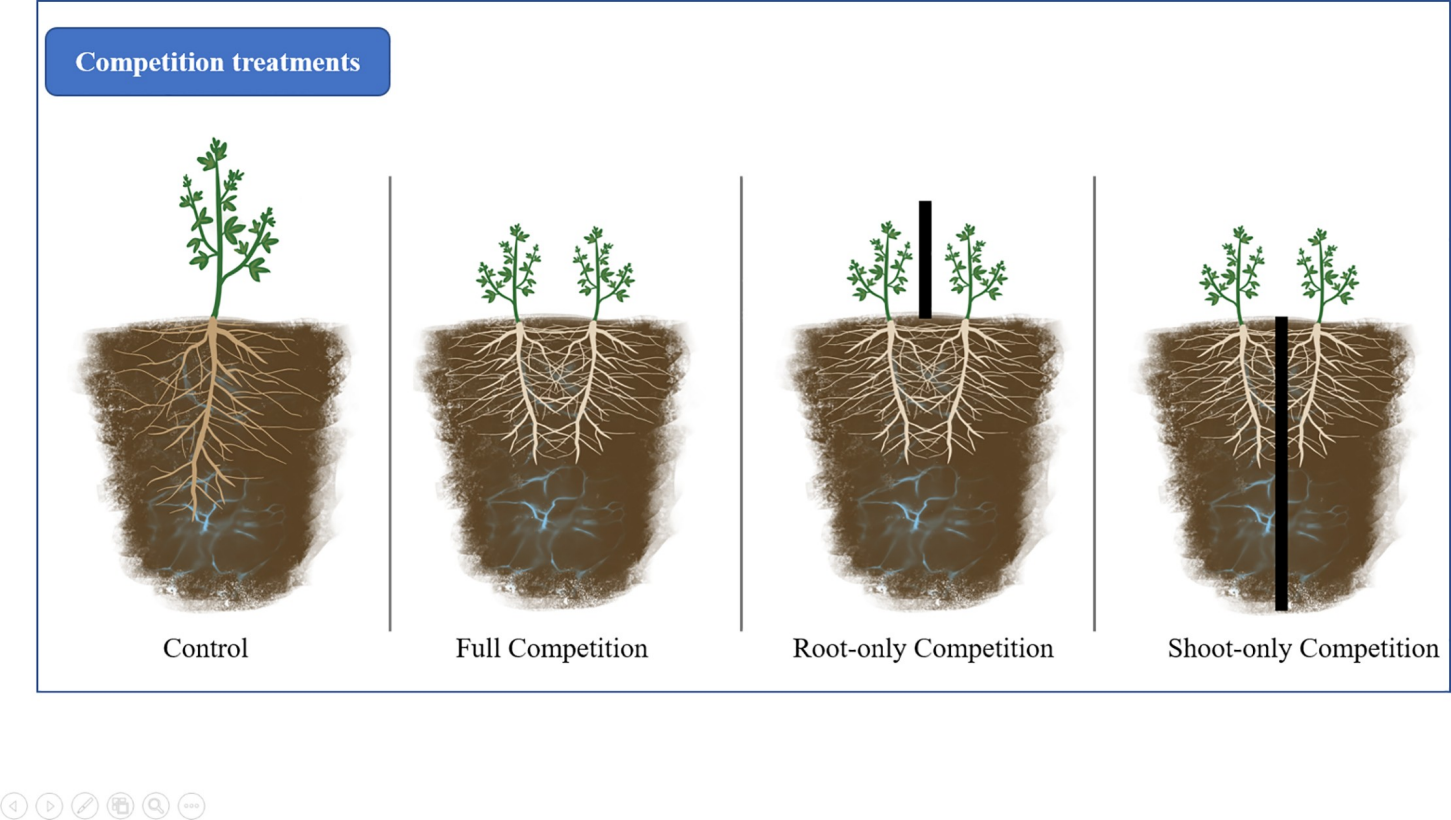
Study treatments. Competition treatments of root-only, shoot-only, full competition and, monoculture of partition studies.

A major question among plant ecologists is to understand plant competition mechanisms and their outcomes from different perspectives. Many contemporary ecological endeavors seek to elucidate the role of competition in community structure, processes, and species coexistence [1–6]. Evidence shows that competition impacts survival, and higher level processes such as community diversity and spatial structure [7,8]. Past work dived deeply into understanding the role of pair-wise species competition on outcomes observed in communities and in field settings [9–12]. But, only a small section of the literature describes the competitive contributions of roots and shoots separately (Fig 1) and their interaction with environmental constraints—which is critical considering the contribution of roots and shoots to ecosystem processes and responses to environmental changes[13–15].

Most competition studies focus on competitive outcomes on shoots. But competitive behaviors resulting from shoot competition, may not influence competitive root responses in the same plant [16], thus the influence and outcome of roots interaction needs specific consideration. Traits can predict competitive ability and performance in environments [17,18], and Kembel & Cahill [19] showed that roots face different environments than shoots leading to variable correlation of above- and belowground traits in response to the environment. A meta-analysis on studies that physically partitioned roots and shoots during competition under nutrient stress found that roots imposed more intense competition than shoots reporting a 42% biomass reduction–indicating intense competition. [20]. An important remaining question is on the role of water in competition.

**Fig 2:**
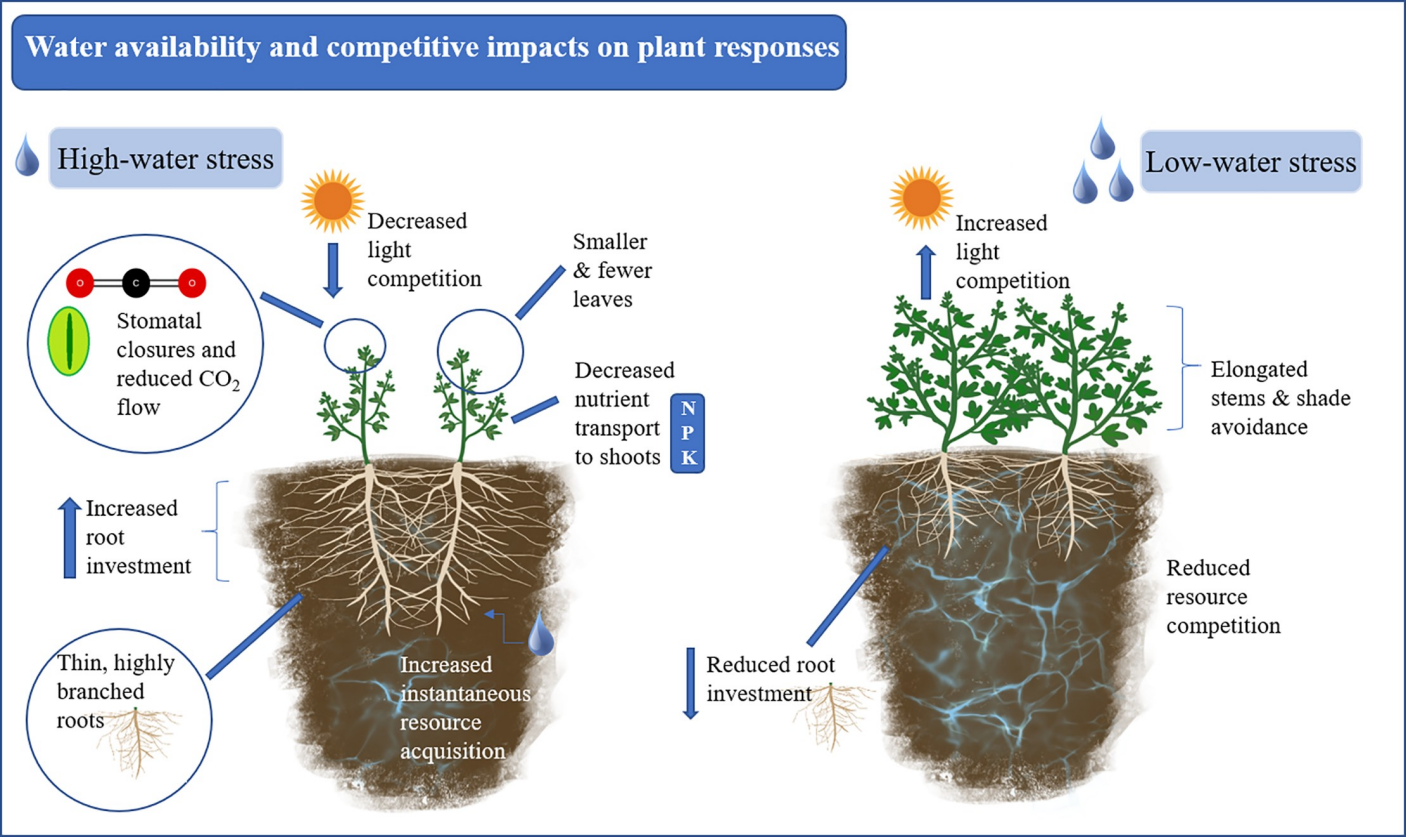
Competition and water stress impacts. Morphological and physiological above- and belowground competitive responses to water availability.

Water is a critical resource that allows plant growth, and related physiological processes such as cell growth and nutrient transport to shoots [21,22]. In cases of low water availability plants can close stomata to limit water loss and CO_2_ capture [23]. Plants can also respond to low water availability by allocating more mass to roots to acquire the limited resource [24,25]. Generally, while water stress reduces plant size, root allocation, branching, length, and uptake increase to maintain soil water capture capacities [26–29] (Fig 2). Conversely, water stress reduces shoot growth, leaf area, new leaf production, and photosynthetic light conversion [27,29–31] (Fig 2). Resulting diminished light interception and metabolic activity aboveground [32], coupled with increased absorptive root area under water stress should intensify competition between roots more than between shoots (e.g. [33]), but the literature presents mixed evidence related to their outcomes.

Despite established patterns of the effects of water stress, water stress intensifies, decreases or produces no measured outcomes on root-only or shoot-only competition (e.g. [34–36]. The different physiological processes of roots and shoots to drought, may reduce resource need. These differing activity levels during drought may also have strong effects on above- compared to belowground performance that may affect the intensity of root and shoot competition in water limited environments. This is critical due to the predicted variable global precipitation patterns and increased regional aridity due to climate change [37]. Environmental constraints such as resource stress, change the intensity of the competition among species [38–41] and low water availability can intensify [42,43] or weaken competition [44].For example, water loss of a nurse shrub due to dry soil reduced mortality in a protégé shrub [45]. Despite the substantial impacts water limitation imposes on competition and survival compared to nutrient stress [46], the literature pool on water and competition is comparatively small so syntheses would advance our knowledge by elucidating patterns.

We conducted a systematic review and meta-analysis to provide resolution on the intensity of root and shoot competition under water stress. We assessed whether roots and shoots impose different competitive intensities in studies that physically partitioning roots and shoots during competition experiments under different water availabilities (Fig 1). We hypothesize that: 1) competitive intensity of root-only, shoot-only, and full competition will differ under varying water availabilities; 2) competitive intensity will differ between low and high water availability treatments; and 3) root competition will differ from shoot competition at varying water availabilities.

## Methods

### Literature search

We sought peer reviewed literature using the ISI Web of Science searching platform. A search was performed on 2 May 2019 of the following title and topic with Boolean terms and wildcard symbols to broaden the search: [(shoot* AND root*) OR (above AND below)] AND [(competit* OR interact*)], topic: “water stress.” Search results were refined by research areas of plant sciences, agriculture, genetics, heredity, forestry, and environmental sciences, and ecology (See S1 Table and S3 Table for study checklists [47]). Citations within relevant articles were searched as well. Abstracts were then evaluated for relevance and kept if they met the following experimental criteria: experimental designs that contained root-only, shoot-only, and or full competition, and a control group (Fig 1), all under a high and low water availability treatments. Authors were contacted for data sharing when essential data were not imputable or extractable.

### Data collection

Studies were included in the analyses if we acquired response variables, standard deviation, and sample sizes, either from the study, the study authors, or from figures. When data were only available in graphics, those data were extracted using the free web-based application WebPlotDigitizer v4.1 [48]. We extracted data from figures from three studies [34,49,50]. Two studies implemented multiple water treatments [51,52], so data from the two extreme treatments were used (highest and lowest water availability). Nutrient treatments were used in some studies, but this was not replicated in all studies nor a target hypothesis so, only data from the lowest nutrient level were utilized. Fixed effects from each study included water treatment (low and high water availability treatments), competition treatment (control, root-only, shoot-only, or full competition) (Fig 2), and focal species nested within study as a random effect.

### Analyses

All analyses were performed in R v3.6.1 [53]. We constructed mixed effects meta-regression models to compare the log response ratio values (lnRR). Models were constructed using the “rma.mv” function in the “metafor” package [54] in R [53]. Models were compared using logliklihood ratio test that used the “anova” function. To test whether water treatments modulated outcomes of the competition treatments, the full model assessed the interaction between water availability levels (low and high availability) and competition treatments (root-only, shoot-only, and full). The reduced models were compared to the full model to determine which explained more variation in plant growth. The reduced models assessed plant growth response to water availability, or plant growth responses to competition treatment, and plant growth responses to the additive effects of competition and water treatments.

The effect sizes lnRR [53]. Log response ratios are the proportional change in treatment groups compared to the control group [55]. They are symmetric around zero and taking the log linearizes the ratio and leads to a generally normal distribution when the treatment mean is not zero [55]. Log response ratios measure the intensity of interactions; negative values denote competition and positive values denote facilitation, while a lnRR of zero denotes no effect of treatment [56]. The lnRR values were calculated in R using the “ROM” measure in the “escalc” function in the “metafor” package [54]. The “ROM” measure underlies the equation:

$$\mathrm{lnRR}=ln\frac{X_{E}}{X_{c}}$$ (1)

Where X_E_ is the biomass mean of treatment group plants compared to the mean of the control group X_C_. Here, the lnRR values were calculated over study and species and compared between root-only, shoot-only, and full competition, as well as water availability levels. The calculated lnRR is the most likely effect size but confidence intervals are important in interpreting meta-analyses outcomes [56]. They indicate how confident one is in the directionality of an effect size and tell the full range of effect size for the treatment [56]. If the lower bound confidence interval overlaps with zero, the results are not statistically significant [56]. The sampling variances of the lnRR were calculated in R using the “escalc” function, and the equation follows Hedges et al. [53]:

$$\frac{{SD}_{E}^{2}}{n_{E}*X_{E}^{2}}+\frac{{SD}_{C}^{2}}{n_{C}*X_{C}^{2}}$$ (2)

Where n_E_ and n_C_ and SD_E_ and SD_C_ are the sample sizes and standard deviations for the experimental and control groups respectively. Standard deviation was not reported in two suitable studies [51,52], but were imputed to reduce publication bias and improve variance estimates compared to when data from an incomplete study are excluded [57]. So, the standard deviation was calculated using F-statistics reported in the original study using Eq 3 (L. Hedges, Personal communication):

$$s=\sqrt{{\left(Y_{C}-Y_{E}\right)}^{-2}*{\left(\frac{F}{\frac{{n_{C}+n}_{E}}{{n_{C}*n}_{E}}}\right)}^{-1}}$$ (3)

where n_C_ and n_E_ are the sample sizes of the control group and treatment group respectively. Additionally, Y_C_ and Y_E_ are the mean values of the control group and treatment group respectively, and s is the imputed standard deviation. Standard deviations were also imputed for one study [51] using a linear regression between sample sizes and pooled standard deviation values of studies with known standard deviation values using the following equation [57]:

$${SD}_{j}={avgX}_{j}*(\sum_{i}^{K}{SD}_{i}/\sum_{i}^{K}{avg\;X}_{i})$$ (4)

where SD_j_ is the standard deviation of the study with missing information and SD_i_ is the standard deviation of samples with full information, X_i_ is the mean of the lnRR of full studies and X_j_ is the mean of the lnRR of the study with missing information. We performed contrasts to test the hypotheses that root competition differed from shoot competition at differing water levels, and the hypothesis that competitive intensity differed between water availability levels. Contrasts were specified in the “anova” function from the “car” package [58]. Finally, we tested for publication bias by performing a Rank Correlation Test for Funnel Plot Asymmetry using the “ranktest” function in the “metafor” package. This helps determine if the observed outcomes and variances are correlated, indicating publication biases [54].

## Results

### Literature search

**Fig 3:**
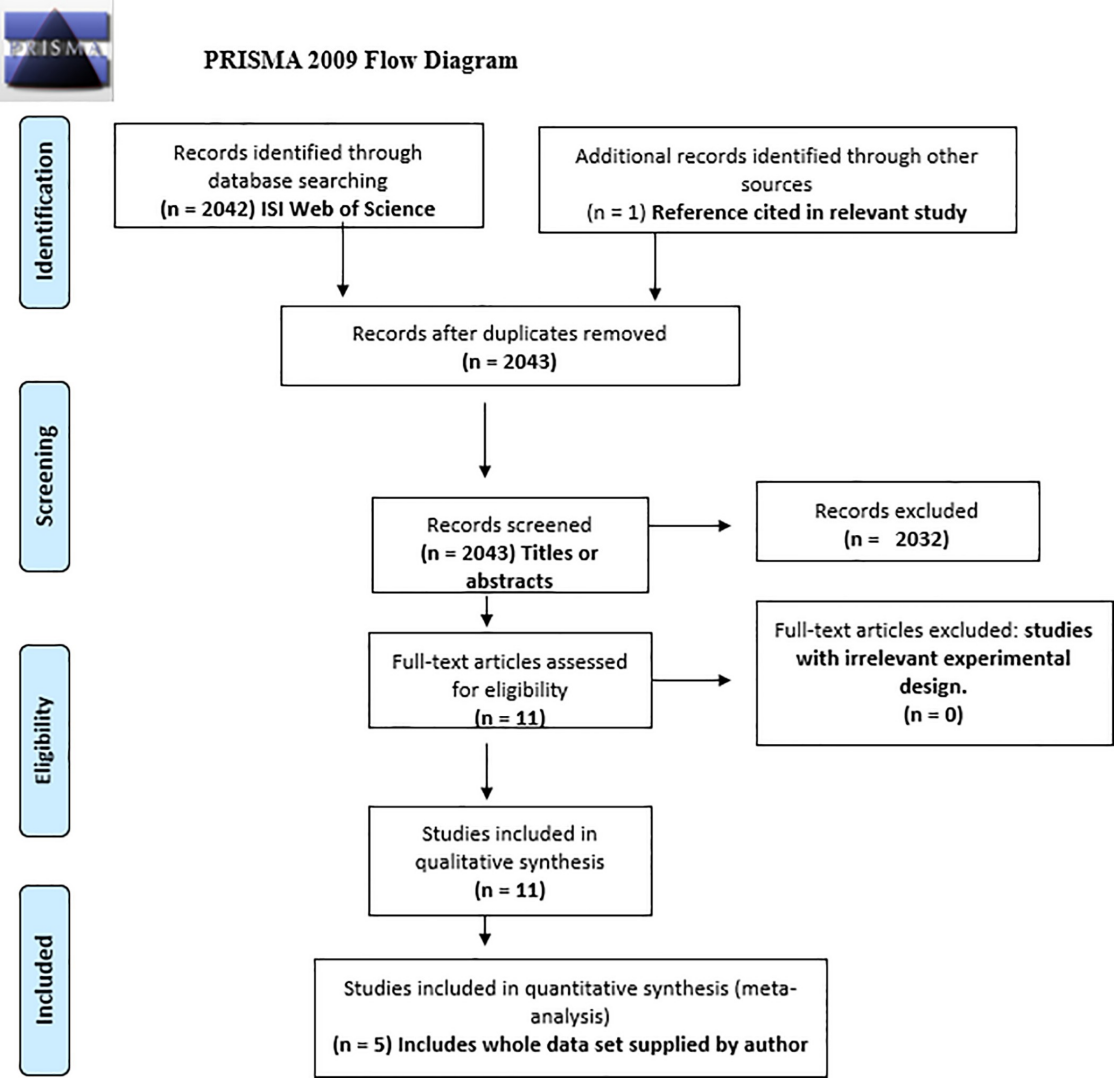
PRISMA flow diagram for study selection. *From*: Moher D, Liberati A, Tetzlaff J, Altman DG, The PRISMA Group (2009). Preferred Reporting Items for Systematic Reviews and Meta-Analyses: The PRISMA Statement. PLoS Med 6(7): e1000097. 10.1371/journal.pmed1000097. **For more information, visit:** www.prisma-statement.org. [61].

The search results yielded 2042 studies (Fig 3).The broad search terms led to many studies that were usual competition experiments that lacked partitions or had suitable methods but manipulated nutrients (see Kiaer et al. [20]) and not water levels, or manipulated no resource. Eleven studies with applicable methods were found. One researcher provided data from her study [36]. Five studies with extractable information were included in the meta-analysis on ten species, containing 106 data points, and 80 lnRR outcome measures (Data for calculations; S2 Table). Data useful for calculating effect sizes and variance were unavailable in figures or through authors in other studies and were excluded from analysis. One excluded study used trees as focal plants [59] while all others utilized herbaceous or shrub species. Furthermore, this study [58] and another [35– also with missing data] used spatial (site differences) and temporal (drought year and rainy season) proxies for water treatments likely introducing heterogeneity and doubts on whether the effect sizes are drawn from the same population–an assumption of fixed effects meta-analytic models [60]. Another study [49] was excluded due to the response measure being shoot to root ratio while all other studies used direct biomass measures. In total, 16 species were represented in all twelve studies published across a 46-year period from 1961–2007 (Table 1).

**Table 1:** Characteristics of studies assessed in this systematic review.

Study	Experimental Setting	Target Species	Response measure	Used in Meta-analysis
Bartelheimer et al. 2010	Outdoor–mesocosm	*Senecio aquaticus; Senecio jacobaea*	Total biomass	Yes
Bornkamm et al. 1975	Setting unknown–pots	*Arrhenatherum elatius; Bromus erectus*	Root biomass	Yes
Lamb et al. 2007	Outdoor–plots	*Artemisia frigida; Chenopodium leptophyllum*	Shoot biomass	Yes
Weigelt et al. 2005	Outdoor–mesocosm	*Carex arenaria; Corynephorus canescens;* *Hieracium pilosella*	Total biomass	Yes
Wilkinson & Gross 1964	Greenhouse–pots	*Trifolium repens*	Total biomass	Yes
Salinger & Bornkamm 1982	Setting unknown–pots	*Arrhenatherum elatius; Bromus erectus*	Shoot:Root ratio	No
Putz and Canham 1992	Outdoor–plots	*Cornus racemosa*	Basal area daily growth rate	No
Dauro & Mohamed-Saleem 1995	Outdoor–mesocosm	*Triticum durum* var. Boolai; *Trifolium quartinianum*	Total biomass	No
Semere & Froud-Williams 2001	Greenhouse–pots	*Zea mays*	Shoot biomass	No
Haugland & Froud-Williams 1999	Greenhouse–pots	*Lolium perenne*	Total biomass	No
Welbank 1961	Outdoor–pots	*Impatiens parviflora*	Biomass growth rate	No

### Interaction outcomes from meta-analysis

**Fig 4:**
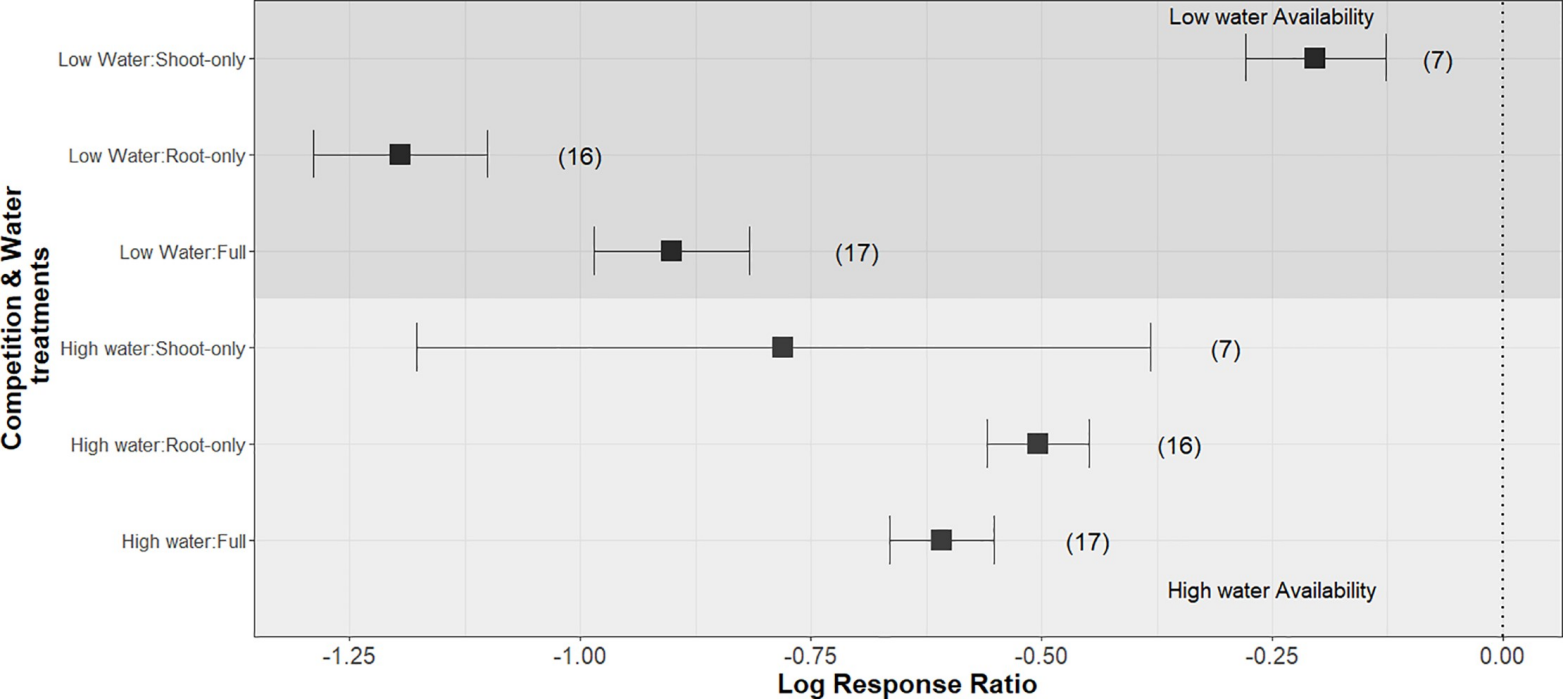
Effects of water availability and competition on plant growth. Meta-estimates (square points) and 95% confidence intervals. Smaller values indicate intense competition, while larger values indicate weaker competition. Sample sizes of lnRR values are in parentheses.

The model that best fit the data included an interaction between competition treatment and water treatments (Q_df = 5_ = 395.5, p < 0.001) (Table 2), whereby competition and water treatments interacted to significantly affect plant growth. Root-only, shoot-only and full competition exhibited different responses to water treatments while opposing competitive outcomes are recorded at low water availability (Fig 4). Shoot-only competition in high water availability resulted in a lnRR of -0.78, while, root-only and full competition are -0.5 and -0.61 respectively, meaning shoot-only competition was on average more intense (Fig 4). Conversely, at low water availability, root-only and full competition treatments resulted in more intense competition (lnRR = -0.9, and lnRR = -1.2, respectively) than shoot-only competition (lnRR = -0.2) (Fig 4).

**Table 2:** Table of model outcomes.

Factor	Df	Q_m_	T^2^	I^2^	P -value
Competition treatment	2	28.7	0.54	98.9%	<0.0001
Water treatment	1	218.1	0.57	99.4%	<0.0001
Competition + water treatment	3	312.6	0.53	98.7%	<0.0001
Competition * water treatment	5	395.5	0.53	98.1%	<0.0001

Q test statistic assess significance of between study variation [55]; T^2^ measures the between study variance; and I^2^ measures variance explained by heterogeneity between studies [54].

Root only-competition significantly differed from shoot-only competition at low water availability (p < 0.0001) and under high water availability (p = 0.04), where root-only competition was more intense under low water availability compared to high water availability. Though there are large confidence intervals for shoot-only competition at high water availability reduces our certainty of the true effect size.

The heterogeneity between studies (Q_m_ on 5 df) is 395.5 indicating that heterogeneity between studies is high and given a Q > 100 we reject the null hypothesis that the variance component is 0 [55]. Large heterogeneity indicates that here are differences between studies and unexplored sources of variation we did not capture in the analyses. This is reinforced by the high I^2^ values (Table 2) denoting that a large part of the variation remains unexplained. Root-only and shoot-only competition had significantly different responses to water treatments (p <0.001) where root-only competition was more intense than shoot-only competition under low water availability and the opposite pattern at high water availability treatments (Fig 4).The overall plant response was only slightly impacted by water availability (p = 0.1). Low water availability caused weaker competition compared to high water availability when aggregated over effect sizes of all treatments. The rank correlation test for funnel plot asymmetry to test for publication bias revealed some correlation between studies (Kendall's tau = 0.153, p = 0.05) indicating publication bias.

### Study Assessments: Competitive outcomes

Welbank [62] is the earliest experiment considered and assessed competition between *Impatiens parviflora* and *Agropyron repens* in pots and only included full competition and shoot-only competition which provides indicative rather than direct impacts of root competition. Full competition under low water availability had a slower growth rate (biomass) than in high water availability and full competition suppressed growth rate more than shoot-only competition, indicating that the inclusion of root intensified competition. In another study, Wilkinson & Gross [51] aimed to understand the role of competition in *Trifolium repens* in stands of *Dactylis glomerata* and introduced *T*. *repens* into stands of *D*. *glomerata* in a greenhouse study where roots and shoots were separated by clear plastic. The biomass of *T*. *repens* at low water availability was highest in full competition followed by root-only competition. Outcomes in low water availability showed that full competition had the greatest mass followed by root-only competition, then by shoot-only competition, indicating that competition was least intense for shoot-only competition.

Bornkamm et al. [52] explored the role of water availability on competition that could pattern distribution of the co-occurring grasses *Arrhenatherum elatus* and *Bromus erectus*. *A*. *elatus* had smaller root mass at low water availability compared to high water availability indicating suppression, and larger roots in shoot-only and full competition treatments under low water availability. *B*. *erectus* had smaller roots in high water availability for root- and shoot-only competition but had larger roots in low water availability showing the opposite pattern. The follow-up study [49] used the same experimental design [52] and found that *B*. *erectus* allocated more mass to shoots under root-only competition and low water availability compared to high water availability. *B*. *erectus* also allocated less to shoots in low water compared to high water availability in both shoot-only and full competition treatments pointing to increased competition. *A*. *elatus* showed a differing response and had lower S:R ratio in low water availability in root-only and full competition, while it had equal S:R in the shoot-only competition for both water levels. Another study [59] assessed methods to curtail tree encroachment into shrub areas and compared the interactions of *Cornus racemosa* on *Acer rubrum* seedlings in a field study using site differences as a proxy for water treatment and trenches with weed cloth and wire to tie shoots. Measuring basal area daily growth rate, the authors found that the growth of *A*. *rubrum* was most suppressed by shoot-only competition—being two times smaller than under root-only competition—meanwhile full competition most suppressed the basal area at the driest site and shoot-only plants had two times the basal area than root-only competition indicating weaker competition in the shoot-only treatment. These site differences also introduce soil property and site history differences that could affect plant growth in addition to treatments imposed.

Two studies utilized partitioning experiments in agricultural systems to understand competition in intercropped systems. Dauro & Mohamed-Saleem [35] evaluated the impacts of competition between intercropped *Triticum durum* var. Boolai and *Trifolium quartinianum* in field plots using wet and dry seasons as a proxy for water treatment and reflective foil and plywood to separate shoots and roots, respectively. In both the dry and wet season shoot-only competition did not significantly affect either species’ biomass, meanwhile root-only competition in the dry season suppressed *T*. *quartinianum* significantly leading to increases in biomass for *T*. *durum* competitors. Semere & Froud-Williams [63] explored ways that intercropping interactions improved yield of *Zea mays* and two pea cultivars with leafy and less-leafy phenotypes in a greenhouse. The authors found that pea cultivar identity and low water availability impacted root-only competition on *Zea Mays*. Both pea cultivar’s growth were not significantly affected by shoot-only competition, while root-only competition and low water availability reduced mass by 43%. These results indicate that root-only competition impacted growth while shoot-only competition had smaller effects, and that water stress and root-only competition suppressed the growth of *Z*. *mays* more than shoot-only competition. Interestingly, pea cultivar competitive intensity in shoot-only treatment did not differ given the differences in leaf phenotype.

Haugland & Froud-Williams [64] explored the role of competition in grassland establishment of established *Lolium perenne* and *Phleum pretense* seedlings in boxes in the greenhouse. The outcomes are not clearly reported likely due to lack of statistically significant findings in competition treatments with water treatments. However, the authors found that low water availability reduced growth of both species and that shoot-only competition from *L*. *perenne* reduced the biomass of *P*. *pratenses* more than root-only competition. Some studies utilized this approach on outdoor settings and mesocosms. Lamb et al. [34] were interested in identifying the role of root-only and shoot-only competition and productivity gradients in Canadian grassland in the field with PVC pipes for root exclusion and plastic netting for shoot exclusion. Focal species were *Artemisia frigida* and *Chenopodium leptophyllum* and neighbors were a mixture of grass and tree species in the natural vegetation. Shoot biomass for *A*. *frigida* under root-only and full competition was similar and smaller than shoot-only competition under both water treatments indicating more intense competition and suppression in these treatments. Shoot biomass in shoot-only competition was smaller with higher water—compared to lower water availability. *C*. *leptophyllum* under low water availability for full and root-only competition had similar shoot mass outcomes, while shoot mass in shoot-only competition treatment was higher. At higher water availability, full competition had the lowest shoot mass mean followed by root-only then shoot-only competition. These results show that competition intensifies when roots interact and under low water availability. The natural vegetation could have potential diversity effects that could influence interaction outcomes though provides a robust comparison of field performance.

Weigelt et al. [36] assessed root allocation in response to competition and resource stress in dune species *Carex arenaria*, *Corynephorus canescens*, and *Hieracium pilosella* in an outdoor sandbox mesocosm calculating competitive intensity from total plant biomass. This study assessed root-only and full competition treatments only and did not report on competition by water treatments responses likely due to the lack of statistical significance. The authors found that competition for all species was generally more intense under low—compared to high water availability. Lastly, one study explored the role root-only or shoot-only competition played in niche segregation of co-occurring species *Senecio aquaticus* and *Senecio jacobea* using *Phleum pratense* competitors in mesocosms under drought and water-logged conditions [50]. *S*. *aquaticus* is adapted to wet soil (e.g. marshes) and had the largest shoot mass in shoot-only competition followed by full, then root-only competition at low water availability. It performed better in high water availability for all treatments, and the competitive hierarchy of low water availably was maintained. *S jacobea* had similar mass in shoot-only and root-only competition treatments and full competition had the smallest mass at low water availability. This species also had the largest shoot mass in shoot-only followed by, full, then root-only competition at low water availability indicating less intense competition with shoot competitors at low water availability.

## Discussion

The impact of increasing drought in a changing climate [65] and ever-present competition have large ramifications for natural plant communities and agricultural systems. Specifically, competition and water stress impacts community membership [3,66] and crop yield [10,67] and has global importance for plant conservation and food security. We demonstrate that water availability significantly modulates competitive outcomes where high water availability intensified shoot-only competition while weakening root-only competition respective to competitive outcomes of low water availability. These study results are important as short-term effects of competition were a top predictor of species’ abundance in the field [68]. This systematic review combines study assessments and a meta-analysis on empirical evidence to reveal competitive patterns and influence future work to advance our knowledge.

### Shoot competition responses to water availability

We show in meta-analysis and in study evaluations that shoot-only competition was more intense under high water availability than in low water availability treatments. Higher aboveground biomass in high water availability treatments may have resulted from plentiful soil resources available for biomass production[27,29–31]. Furthermore, greater aboveground mass could be in response to light competition for shade avoidance responses denoting intensified competition through imposing shade [69,70]. From a community perspective, research suggests that light competition is important in ecosystems with high aboveground productivity [71] and thus aboveground competition can impact patterns of community diversity and dynamics [72].

To the contrary, the weakest competitive treatment was shoot-only competition in low water availability. Water stress is known to limit plant growth leading to a reduction in leaf area which limits shading and light competition that an individual can impose on its neighbor [63]. Results of the meta-analysis showed that competition weakens at low water availability when shoot competition is included, and seem to agree with the stress gradient hypothesis which notes that facilitation and weak competitive interactions may dominate at high-stress levels compared to low-stress [44,73]. Weak competitive interactions could be a result of plants allocating less mass aboveground or slowing metabolic activity aboveground for survival and defense under stressful conditions [32]. This is interesting given that competition in dry environments is high, though thought to be concentrated belowground [74], however, we clearly demonstrate that when shoot competition is considered alone water availability is a key factor modulating its intensity and this needs exploration in different biomes.

### Root responses to water availability

Root-only competition was weaker at high water availability than low water availability but was the most intense competition group of this study at low water availability. This suggests that higher water supply weakens belowground competition and shows different patterns to shoot-only competition. These results are in line with Lamb et al. [34], but counter Bartelheimer et al. [50] who showed competitive suppression in root-only treatments under high water supply. On the other hand, root-only competition was the most intense competition treatment under low water availability. Intense root competition may be driven by roots responding to water stress by increasing root allocation and intensity of soil exploration resulting in increased nutrients and water uptake [24,25,75,76]. High root biomass and root length production are known to induce intense competition between plants [77] and these morphological changes in response to water stress likely also increase competition due to reduced resources [10,78]. Research suggests that root competition is more intense in dry environments where productivity is concentrated belowground [74,79] and root-only competition was more intense than shoot-only competition under low water availability. These results along with meta-analytic findings of Kiaer et al. [20] on nutrients indicate that when soil resources are limited, root competition is more intense than shoot competition. Despite this strong evidence of a positive effect of water shortage on the root competition we may expect conflicting responses when species evolve in differing environments, though more studies are needed to better assess this hypothesis.

### Whole plant outcomes and implications

The results of studies reviewed highlight the variability in species response to low water availability but generally are in line with the findings of this meta-analysis that root-only competition differs from shoot-only competition. But the contrasting results between shoot-only, root-only, and full competition suggest that the contributions of root and shoot competition are not additive. Rajaniemi et al. [38] showed that root-only competition experimental assemblages resulted in lower species diversity compared to shoot-only competition assemblages. Also, Lamb et al. [80] showed shoot competition negatively impacted community evenness but was through indirect increases in competitive root responses. While aboveground competition has documented impacts on community structure [81], root competition also has strong and apparent consequences for plant communities. Because we see contrasting outcomes in root-only and shoot-only competition, researchers should increase the assessment of belowground ecology to draw more accurate conclusions about competition particularly if environmental constraints would lead to a shift in biomass allocation [82].

### Study limitations

These results show important interactions between plant competition and water availability. The fixed effects used in these models significantly explained variation in effect sizes but including other effects such as target species life history, non-target life-history, and experimental setting may reduce residual heterogeneity. Given the small number of studies, these factors could not be reliably tested without replication. Other sources of variation were in the differences in materials used to partition plants (e.g. mesh vs. solid aboveground dividers) and implementation of water stress where amounts that were considered “high” and “low” differed by study. Additionally, the adaptations of target species could have influenced competitive outcomes and responses to water stress. For example, Bartelheimer et al. [50] used *Senecio aquaticus*–a wetland adapted species–which performed poorer than the terrestrial congener in low water availability.

Five studies ignored the role of intraspecific competition in the set-up and had focal plants interact with conspecifics both above and belowground. Given that many species compete more intensely with conspecifics than heterospecifics [83] this could impact the outcomes of competitive intensity recorded. Additionally, considering proper comparison groups is important for quantifying the effect of a treatment. Monoculture groups with root-only, shoot-only, full treatments under all applied water treatments serve as appropriate controls for partition studies.

Finally, we excluded several known suitable studies from the meta-analysis due to missing information introducing publication bias [84]. More studies in this area are needed particularly to provide resolution for whether plants alter allocation in response to the source of below or aboveground competition, shedding light on long-posited hypotheses [76]. The results of relevant treatments in suitable studies were likely not reported due to lack of significance, introducing selective reporting bias [84]. Authors should publish full study results related to original hypotheses presented and parameters (e.g. sample size, responses, measures of variability) for future synthesis and knowledge advancement.

### Conclusions

The intensity of root-only and shoot-only competition showed opposing trends under differing water availability. Our results show that roots have major implications in competitive outcomes for plants when soil resource are limited. Importantly, if we only record aboveground responses to water stress or competition, we may conclude weak competition or facilitation when belowground responses may reveal contrasting evidence. Future research should tie in the role that root and shoot competition have on species coexistence in plant communities.

## Supporting information

S1 Table Checklist. PRISMA checklist.
(DOCX)

S2 Table Study data. Study dataset used to calculate effect sizes (lnRR) and sampling variances.
(DOCX)

S3 Table Koricheva & Gurevitch (2014) meta-analysis checklist. (DOCX)

S1 Fig Meta-estimates by study. (TIFF)
