# Supplementary material for: Root and shoot competition lead to contrasting competitive outcomes under water stress: A systematic review and meta-analysis
Source: PLoS One. 2019 Dec 11;14(12):e0220674. doi: 10.1371/journal.pone.0220674 (PMC6905553; doi:10.1371/journal.pone.0220674)
Supplement: S1 Table — PRISMA checklist. (DOCX) [file pone.0220674.s001.docx]

| **Section/topic** | **#** | **Checklist item** | **Reported on page #** |
| --- | --- | --- | --- |
| **TITLE** | | |  |
| Title | 1 | Root and shoot competition lead to contrasting competitive outcomes under water stress: A Meta-analysis | 1 |
| **ABSTRACT** | | |  |
| Structured summary | 2 | **Background:** Competition is a critical process that shapes plant communities and interacts with environmental constraints. Though important to natural communities and agricultural systems, there are surprising knowledge gaps related to mechanisms that belie those processes: the contribution of different plant parts on competitive outcomes and the effect of environmental constraints on these contributions.  **Objective:** Studies that partition competition into root-only and shoot-only interactions assess whether plant parts impose different competitive intensities using physical partitions and serve as an important way to fill knowledge gaps. Given predicted drought escalation due to climate change, we focused meta-analytic techniques on the effects of water supply and competitive outcomes.  **Methods:** We searched Web of Science for peer-reviewed studies and found 2042 results. From which six suitable studies with 92 effect sizes on 10 species were identified to test these effects.  **Results:** Water availability and competition treatment (root-only, shoot-only, and full plant competition) significantly interact to affect plant growth responses (p < 0.0001). Root-only and full plant competition are more intense in low water availability conditions than shoot-only competition. Shoot-only competition in high-water availability was the most intense showing the opposite pattern. These results also show that the intensity of full competition is similar to root-only competition and that low-water availability intensifies root competition while weakening shoot competition.  **Conclusions:** These results emphasize the importance of root competition and these patterns of competition may shift in a changing climate, creating further urgency for further filling knowledge gaps to address issues of drought on plant interactions and communities. | 2 |
| **INTRODUCTION** | | |  |
| Rationale | 3 | A major question among plant ecologists is to understand plant competition mechanisms and their outcomes from different perspectives. Many contemporary ecological endeavors seek to elucidate the role of competition in community structure, processes, and species coexistence [1–6]. Evidence shows that competition impacts survival, and higher level processes such as community diversity and spatial structure [7,8] Past work dived deeply into understanding the role of pair-wise species competition on outcomes observed in communities and in field settings [9–12]. But, only a small section of the literature describes the competitive contributions of roots and shoots separately (Fig. 1) and their interaction with environmental constraints - which is critical considering the contribution of roots and shoots to ecosystem processes and responses to environmental changes[13–15].  Most competition studies focus on competitive outcomes on shoots. But competitive behaviors resulting from shoot competition, may not influence competitive root responses in the same plant [16], thus the influence and outcome of roots interaction needs specific consideration. Traits can predict competitive ability and performance in environments [17,18], and Kembel & Cahill [19] showed that roots face different environments than shoots leading to variable correlation of above- and belowground traits in response to the environment. A meta-analysis on studies that physically partitioned roots and shoots during competition under nutrient stress found that roots imposed more intense competition than shoots reporting a 42% biomass reduction – indicating intense competition. [20]. A critical remaining question is on the role of water in competition.  Water is a critical resource that allows plant growth, and related physiological processes such as cell growth and nutrient transport to shoots [21,22]. In case of low water availability plants can close stomata to limit water loss and CO_2_ capture [23] . They can also respond to water stress by allocating more mass to roots to acquire the limited resource [24,25]. Generally, while water stress reduces plant size, root allocation, branching, length, and uptake, increase to maintain soil water capture capacities [26–29] (Fig. 2). Conversely, water stress reduces shoot growth, leaf area, new leaf production, and photosynthetic light conversion [27,29–31] (Fig. 2). Resulting diminished light interception and metabolic activity aboveground [32], coupled with increased absorptive root area under water stress should intensify competition between roots more than between shoots (e.g. [33]), but the literature presents mixed evidence related to their outcomes.  Despite established patterns of individual effects of water stress, water stress intensifies, decreases or produces no measured outcomes on root-only or shoot-only competition (e.g. [34–36]. The different physiological processes of roots and shoots to drought, may reduce resource acquisition need. These differing activity levels during drought may also have strong effects on above- compared to belowground performance that may affect the intensity of root and shoot competition in water limited environments. This is critical due to the predicted variable global precipitation patterns and increased regional aridity due to climate change [37]. Environmental constraints such as resource stress change the intensity of the competition among species [38–41]. For example, low water availability can intensify [42,43] or weaken competition [44] and, for example, water loss of a nurse shrub due to dry soil reduced mortality in a protégé shrub [45]. Despite the substantial impacts water limitation imposes on competition and survival compared to nutrient stress [46], the literature pool on water and competition is comparatively small so synthesis would advance our knowledge by elucidating patterns. | 3-5 |
| Objectives | 4 | We conducted a meta-analysis to provide resolution on the intensity of root and shoot competition under water stress. We assessed whether roots and shoots impose different competitive intensities in studies that physically partitioning roots and shoots during competition experiments under different water availabilities (Fig. 1). We hypothesize that: 1) competitive intensity of root-only, shoot-only, and full competition will differ under varying water availability; 2) competitive intensity will differ between low – and high-water stress treatments; and 3) root competition will differ from shoot competition at varying water availabilities. | 5 |
| **METHODS** | | |  |
| Protocol and registration | 5 | We sought peer reviewed literature using the ISI Web of Science searching platform. A search was performed on 2 May 2019 | 6 |
| Eligibility criteria | 6 | Criteria: experimental designs that contained root-only, shoot-only, and or full competition, and a control group (Fig. 1), all under a high- and low-water availability treatments. Weigelt et al. [36] lacked a shoot competition treatment but was included here. | 6 |
| Information sources | 7 | ISI Web of Science, contact with study authors to identify additional data. | 6 |
| Search | 8 | We used Boolaen terms to broaden the search: [(shoot* AND root*) OR (above AND below)] AND [(competit* OR interact*)], topic: “water stress.” Search results were refined by research areas of plant sciences, agriculture, genetics, heredity, forestry, and environmental sciences, and ecology. | 6 |
| Study selection | 9 | Abstracts were then evaluated for relevance and read if meet criteria | 6 |
| Data collection process | 10 | Studies were included in the analyses if we acquired response variables, standard deviation, and sample sizes, either from the study, the study authors, or from figures. When data were only available in graphics, those data were extracted from figures using the free web-based application WebPlotDigitizer v3.9 [47]. We extracted data from figures from three studies [34,48,49]. | 6 |
| Data items | 11 | We collected additional data such as study location, setting species, water treatment | 6 |
| Risk of bias in individual studies | 12 | Five suitable studies were not analyzed due to missing data. These studies are summarized in the discussion to compare their outcomes to studies analyzed. Standard deviations were imputed on three studies to include here. This reduces publication bias and improves variance estimates compared to when data from an incomplete study are excluded [56]. | 8-9 |
| Summary measures | 13 | Log response ratio, sampling variance | 7-8 |
| Synthesis of results | 14 | Q, I^2^, T^2^ and sigma are reported herein | 11 |

Page 1 of 2

| **Section/topic** | **#** | **Checklist item** | **Reported on page #** |
| --- | --- | --- | --- |
| Risk of bias across studies | 15 | Factors across each study were held constant such as water level (lowest or highest levels are included when there were multiple levels included). Different species were used in many of the studies and phylogenetic independence may lead to correlation between species. | 6 |
| Additional analyses | 16 | Contrasts were performed to compare specific groups within treatments. | 9 |
| **RESULTS** | | |  |
| Study selection | 17 | 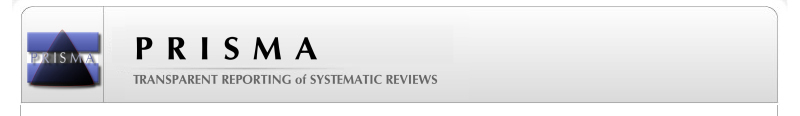  **PRISMA 2009 Flow Diagram**   Included Additional records identified through other sources (n = 1 ) **Reference cited in relevant study**  Records after duplicates removed **(n = 2043 )**  Records identified through database searching **(n = 2042)** **Web of Science** Identification Records screened **(n = 2043)** **Titles or abstracts**  Records excluded **(n = 2032)** Screening Full-text articles excluded: **studies with irrelevant experimental design.**  **(n = 0)**  Full-text articles assessed for eligibility **(n = 11)** Eligibility Studies included in qualitative synthesis **(n = 11)**  Studies included in quantitative synthesis (meta-analysis) **(n = 5)** **Includes whole data set supplied by author** | SI Fig 1 |
| Study characteristics | 18 | Bartelheimer M, Gowing D, Silvertown J. Explaining hydrological niches: The decisive role of below-ground competition in two closely related Senecio species. J Ecol. 2010;98: 126–136. doi:10.1111/j.1365-2745.2009.01598.x  Bornkamm R, Salinger S, Strehlow H. Productivity and Chemical Constituents of two Grasses under Pure and Mixed Cultivations. Flor Biodivers. 1975;164: 437–448.  Dauro D, Mohamed-Saleem M. Shoot and root interactions in intercopped wheat and clover. Trop Agric. 1995;72: 170–172.  Lamb EG, Shore BH, Cahill JF. Water and nitrogen addition differentially impact plant competition in a native rough Fescue grassland. Plant Ecol. 2007;192: 21–33. doi:10.1007/sll258-006-9222-4  Haugland E, Froud-Williams R. Improving grasslands: the influence of soil moisture and nitrogen fertilization on the establishment of seedlings. J Appl Ecol. 1999;36: 263–270.  Putz F, Canham C. Mechanisms of arrested succession in shrublands: root and shoot competition between shrubs and tree seedlings. For Ecol Manage. 1992;49: 267–275.  Salinger S, Bornkamm R. Production of organic matter and interference of two grasses at different levels of water supply. Agro-Ecosystems. 1982;7: 277–292.  Semere T, Froud-Williams RJ. The effect of pea cultivar and water stress on root and shoot competition between vegetative plants of maize and pea. J Appl Ecol. 2001;38: 137–145. doi:10.1046/j.1365-2664.2001.00570.x  Weigelt A, Steinlein T, Beyschlag W. Competition among three dune species: the impact of water availability on below- ground processes. Plant Ecol. 2005;176: 57–68.  Welbank PJ. A Study of the Nitrogen and Water Factors in Competition with Agropyron repens (L.) Beauv. Ann Bot. 1961;25: 116–137. doi:10.1093/oxfordjournals.aob.a083737  Wilkinson S, Gross C. Competition for light, soil moisture and nutrients during Landino clover establishment in Orchardgrass Sod. Agron J. 1964;56: 389–392. | 9 |
| Risk of bias within studies | 19 | The sample size within some studies is small and may introduce bias. For example, Bartelheimer et al. 2010 had the smallest treatment sample size of n = 3. The rank correlation test for funnel plot asymmetry to test for publication bias revealed low and non-significant correlation between studies (Kendall's tau = 0.153, p = 0.045) indicating publication bias. | SI Table 1 |
| Results of individual studies | 20 | The model that best fit the data included an interaction between competition treatment and water treatments (Q_df = 5_ = 395.5, p < 0.001) (Table 2), whereby competition and water treatments interacted to significantly affect plant growth. Root-only, shoot-only and full competition exhibited different responses to water treatments (Fig. 3). Root-only (-45%) and full (-53%) competition at low water availability was more intense than shoot-only (-14%) competition, while root-only (-51%) and full (-51%) competition similarly lead to similar mass suppression whereas shoot-only (-36%) competition had the most intense competition outcome though was less suppressive under high water availability.  Root only-competition significantly differed from shoot-only competition at low water availability (p = 0.0004) and under high water availability (p < 0.0001), where root-only competition was more intense under low water availability compared to high water availability. Though there are large confidence intervals for shoot-only competition at high water availability reduces our certainty of the true effect size.  The heterogeneity between studies (Q_m_ on 5 df) is 395.5 indicating that heterogeneity between studies is high (given a Q > 100 we reject the null hypothesis that the variance component is 0 [53]) and there are differences between studies and unexplored sources of variation we did not capture in the analyses. This is reinforced by the high I^2^ values (Table 2) denoting that a large part of the variation remains unexplained. Root-only and shoot-only competition had significantly different responses to water treatments (p <0.001) where root-only competition was more intense than shoot-only competition under low water availability and the opposite pattern at high water availability treatments (Fig. 3).The overall plant response was not significantly impacted by water availability (p = 0.1). Low water availability caused slightly weaker competition compared to compared to high water availability when aggregated over effect sizes of all treatments. The rank correlation test for funnel plot asymmetry to test for publication bias revealed low and non-significant correlation between studies (Kendall's tau = 0.153, p = 0.045) indicating publication bias. | SI Table 1, p 10-11 |
| Synthesis of results | 21 | We found that shoot-only competition was more intense under high-water availability than in low-water availability treatments. To the contrary, the weakest competitive treatment was shoot-only competition in low-water availability. Root-only competition was weaker at high-water availability. Furthermore, root-only competition was more intense than shoot-only competition under low-water availability. | 10-11 |
| Risk of bias across studies | 22 | Three suitable studies were not included in the analysis which influences the risk of publication bias here and the rank correlation test for funnel plot asymmetry to test for publication bias revealed low and non-significant correlation between studies (Kendall's tau = 0.153, p = 0.045) indicating publication bias | 16 |
| Additional analysis | 23 | The overall plant response was not significantly impacted by water availability (p = 0.1). | 11 |
| **DISCUSSION** | | |  |
| Summary of evidence | 24 | The impact of increasing drought in a changing climate [60] and ever-present competition have large ramifications for natural plant communities and agricultural systems. Specifically, competition and water stress impacts community membership [3,61] and crop yield [10,62] and has global importance for plant conservation and food security. We demonstrate that water availability significantly modulates competitive outcomes where high-water availability intensified shoot-only competition while weakening root-only competition and the opposite patterns for low water availability. These results are important as short-term effects of competition were a top predictor of species’ abundance in the field [63]. This meta-analysis combines empirical evidence to reveal competitive patterns and influence future work to advance our knowledge.  Given the climate change outcomes of increased drought leading to increased root allocation [25] this may have important competition-mediated community outcomes. We may see increases in root competition for water in communities (sensu [33], [25]) that lead to plant diversity loss from drought [78]. But more research is needed to assess these outcomes and in different biomes. Because we see contrasting outcomes in root-only and shoot-only competition, researchers should increase the assessment of belowground ecology to draw more accurate conclusions about competition particularly if environmental constraints would lead to a shift in biomass allocation [79]. | 12,14 |
| Limitations | 25 | These results show important interactions between plant competition and water availability. The fixed effects used in these models significantly explained variation in effect sizes but including other effects such as target species life history, non-target life-history, and experimental setting may reduce residual heterogeneity. Given the small number of studies, these factors could not be reliably tested without replication. Other sources of variation were in the differences in materials used to partition plants (e.g. mesh vs. solid aboveground dividers) and implementation of water stress where amounts that were considered “high” and “low” differed by study. Additionally, the adaptations of target species could have influenced competitive outcomes and responses to water stress. For example, Bartelheimer et al. [49] used *Senecio aquaticus* – a wetland adapted species – which performed poorer than the terrestrial species in low water availability. Finally, there were known suitable studies that we excluded due to missing information. Authors should publish robust study results and parameters (e.g. sample size, responses, measures of variability) for future synthesis and knowledge advancement. | 15-16 |
| Conclusions | 26 | The intensity of root-only and shoot-only competition showed opposing trends under differing water availability. Our results show that roots have major implication in competitive outcomes for plants when soil resource are limited. This suggests that root-dominated interactions should make coexistence more difficult and lead to more growth suppression in case of water shortage. Importantly, if we only record aboveground responses to water stress or competition we may conclude weak competition when belowground responses may reveal contrasting evidence. Future research should tie in the role that root and shoot competition has on species coexistence in plant communities. | 16 |
| **FUNDING** | | |  |
| Funding | 27 | This study was not funded directly, though A. Foxx is supported by the Robert Hevey and Constance M. Filling PhD Fellowship | NA |

*From:*  Moher D, Liberati A, Tetzlaff J, Altman DG, The PRISMA Group (2009). Preferred Reporting Items for Systematic Reviews and Meta-Analyses: The PRISMA Statement. PLoS Med 6(7): e1000097. doi:10.1371/journal.pmed1000097

For more information, visit: **www.prisma-statement.org**.
