# Supplementary material for: Root and shoot competition lead to contrasting competitive outcomes under water stress: A systematic review and meta-analysis
Source: PLoS One. 2019 Dec 11;14(12):e0220674. doi: 10.1371/journal.pone.0220674 (PMC6905553; doi:10.1371/journal.pone.0220674)
Supplement: S2 Table — Study dataset used to calculate effect sizes (lnRR) and sampling variances. (DOCX) [file pone.0220674.s002.docx]

**SI Table 2.** Data used for meta-analysis

| Study | Target_species | Water_trt | Comp_trt | N | Cntrl_Mean | Cntrl_STD | notes | LRR | LRR_var |
| --- | --- | --- | --- | --- | --- | --- | --- | --- | --- |
| Bartelheimer et al. 2010 | *Senecio acquaticus* | Ambient | Shoot_Comp | 3 | 7.857143 | 1.254902 |  | 0.00905 | 0.008797 |
| Bartelheimer et al. 2010 | *Senecio acquaticus* | Ambient | Root_Comp | 3 | 7.857143 | 1.254902 |  | -0.33103 | 0.014943 |
| Bartelheimer et al. 2010 | *Senecio acquaticus* | Ambient | Full_Comp | 3 | 7.857143 | 1.254902 |  | -0.96281 | 0.041311 |
| Bartelheimer et al. 2010 | *Senecio acquaticus* | Stress | Shoot_Comp | 3 | 2.630332 | 11.7532 |  | -0.11441 | 6.728362 |
| Bartelheimer et al. 2010 | *Senecio acquaticus* | Stress | Root_Comp | 3 | 2.630332 | 11.7532 |  | -0.83833 | 7.493748 |
| Bartelheimer et al. 2010 | *Senecio acquaticus* | Stress | Full_Comp | 3 | 2.630332 | 11.7532 |  | -0.47542 | 7.490581 |
| Bartelheimer et al. 2010 | *Senecio jacobea* | Ambient | Shoot_Comp | 3 | 2.928571 | 1.254902 |  | -0.21706 | 0.064526 |
| Bartelheimer et al. 2010 | *Senecio jacobea* | Ambient | Root_Comp | 3 | 2.928571 | 1.254902 |  | -0.76913 | 0.172534 |
| Bartelheimer et al. 2010 | *Senecio jacobea* | Ambient | Full_Comp | 3 | 2.928571 | 1.254902 |  | -1.31568 | 0.539495 |
| Bartelheimer et al. 2010 | *Senecio jacobea* | Stress | Shoot_Comp | 3 | 2.843602 | 6.823529 |  | 0.161268 | 1.955372 |
| Bartelheimer et al. 2010 | *Senecio jacobea* | Stress | Root_Comp | 3 | 2.843602 | 6.823529 |  | 0 | 2.053518 |
| Bartelheimer et al. 2010 | *Senecio jacobea* | Stress | Full_Comp | 3 | 2.843602 | 6.823529 |  | -1.12393 | 4.533832 |
| Bornkamm et al. 1975 | *Arrhenatherum elatius* | Ambient | Full_Comp | 7 | 1.72 | 0.023782 | std imputed from F = 4.44 | -0.07859 | 8.89E-05 |
| Bornkamm et al. 1975 | *Arrhenatherum elatius* | Ambient | Root_Comp | 7 | 1.72 | 0.023782 | std imputed from F = 4.45 | 0.051003 | 5.01E-05 |
| Bornkamm et al. 1975 | *Arrhenatherum elatius* | Ambient | Shoot_Comp | 7 | 1.72 | 0.023782 | std imputed from F = 4.46 | -0.26469 | 0.000873 |
| Bornkamm et al. 1975 | *Arrhenatherum elatius* | Stress | Full_Comp | 7 | 1.77 | 0.209708 | std imputed from F = 4.48 | -0.11355 | 0.038443 |
| Bornkamm et al. 1975 | *Arrhenatherum elatius* | Stress | Root_Comp | 7 | 1.77 | 0.209708 | std imputed from F = 4.49 | 0 | 0.002005 |
| Bornkamm et al. 1975 | *Arrhenatherum elatius* | Stress | Shoot_Comp | 7 | 1.77 | 0.209708 | std imputed from F = 4.50 | -0.23451 | 0.002649 |
| Bornkamm et al. 1975 | *Bromus erectus* | Ambient | Full_Comp | 7 | 1.47 | 0.004076 | std imputed from F = 4.52 | -0.83155 | 0.015497 |
| Bornkamm et al. 1975 | *Bromus erectus* | Ambient | Root_Comp | 7 | 1.47 | 0.004076 | std imputed from F = 4.53 | -0.3557 | 0.000634 |
| Bornkamm et al. 1975 | *Bromus erectus* | Ambient | Shoot_Comp | 7 | 1.47 | 0.004076 | std imputed from F = 4.54 | -0.74194 | 0.01115 |
| Bornkamm et al. 1975 | *Bromus erectus* | Stress | Full_Comp | 7 | 0.76 | 0.014552 | std imputed from F = 4.56 | 0.243978 | 0.000484 |
| Bornkamm et al. 1975 | *Bromus erectus* | Stress | Root_Comp | 7 | 0.76 | 0.014552 | std imputed from F = 4.57 | 0.254234 | 0.002356 |
| Bornkamm et al. 1975 | *Bromus erectus* | Stress | Shoot_Comp | 7 | 0.76 | 0.014552 | std imputed from F = 4.58 | 0.303996 | 0.000685 |
| Lamb et al. 2007 | *Artemesia frigida* | Ambient | Full_Comp | 7 | 0.052497 | 0.015602 |  | -1.31759 | 4.079984 |
| Lamb et al. 2007 | *Artemesia frigida* | Ambient | Root_Comp | 7 | 0.052497 | 0.015602 |  | -1.75399 | 0.619933 |
| Lamb et al. 2007 | *Artemesia frigida* | Ambient | Shoot_Comp | 7 | 0.052497 | 0.015602 |  | -0.81214 | 0.02186 |
| Lamb et al. 2007 | *Artemesia frigida* | Stress | Full_Comp | 7 | 0.09284 | 0.058501 |  | -2.12832 | 23.19363 |
| Lamb et al. 2007 | *Artemesia frigida* | Stress | Root_Comp | 7 | 0.09284 | 0.058501 |  | -2.30535 | 0.393603 |
| Lamb et al. 2007 | *Artemesia frigida* | Stress | Shoot_Comp | 7 | 0.09284 | 0.058501 |  | -0.71488 | 0.058992 |
| Lamb et al. 2007 | *Chenopodium leptophyllum* | Ambient | Full_Comp | 7 | 0.049406 | 0.043016 |  | -2.1814 | 27.57345 |
| Lamb et al. 2007 | *Chenopodium leptophyllum* | Ambient | Root_Comp | 7 | 0.049406 | 0.043016 |  | -1.00807 | 0.110764 |
| Lamb et al. 2007 | *Chenopodium leptophyllum* | Ambient | Shoot_Comp | 7 | 0.049406 | 0.043016 |  | -0.31399 | 0.14623 |
| Lamb et al. 2007 | *Chenopodium leptophyllum* | Stress | Full_Comp | 7 | 0.092578 | 0.116589 |  | -1.56994 | 7.576427 |
| Lamb et al. 2007 | *Chenopodium leptophyllum* | Stress | Root_Comp | 7 | 0.092578 | 0.116589 |  | -1.603 | 0.373681 |
| Lamb et al. 2007 | *Chenopodium leptophyllum* | Stress | Shoot_Comp | 7 | 0.092578 | 0.116589 |  | 0.066103 | 0.22926 |
| Weigelt et al. 2005 | *Carex arenaria* | Ambient | Full_Comp | 9 | 4.596222 | 3.390814 | CaCa Ambient Full_comp_monoculture | -0.52841 | 0.119402 |
| Weigelt et al. 2005 | *Carex arenaria* | Ambient | Root_Comp | 9 | 5.530556 | 3.387683 | CaCa Ambient Root_comp_monoculture | -0.36864 | 0.081101 |
| Weigelt et al. 2005 | *Carex arenaria* | Stress | Full_Comp | 8 | 4.202444 | 3.163751 | CaCa Stress Full_comp_monoculture | -1.15927 | 0.124504 |
| Weigelt et al. 2005 | *Carex arenaria* | Stress | Root_Comp | 8 | 3.110444 | 1.496866 | CaCa Stress Root_comp_monoculture | 0.089671 | 0.127769 |
| Weigelt et al. 2005 | *Carex arenaria* | Ambient | Full_Comp | 9 | 4.596222 | 3.390814 | CaCc Ambient Full_comp | -2.1408 | 0.232606 |
| Weigelt et al. 2005 | *Carex arenaria* | Ambient | Root_Comp | 9 | 5.530556 | 3.387683 | CaCc Ambient Root_comp | -2.58549 | 0.071626 |
| Weigelt et al. 2005 | *Carex arenaria* | Stress | Full_Comp | 9 | 4.202444 | 3.163751 | CaCc Stress Full_comp | -2.84035 | 0.09657 |
| Weigelt et al. 2005 | *Carex arenaria* | Stress | Root_Comp | 9 | 3.110444 | 1.496866 | CaCc Stress Root_comp | -2.61074 | 0.071843 |
| Weigelt et al. 2005 | *Carex arenaria* | Ambient | Full_Comp | 9 | 4.596222 | 3.390814 | CaHp Ambient Full_comp | -0.99271 | 0.093416 |
| Weigelt et al. 2005 | *Carex arenaria* | Ambient | Root_Comp | 9 | 5.530556 | 3.387683 | CaHp Ambient Root_comp | -1.22069 | 0.083925 |
| Weigelt et al. 2005 | *Carex arenaria* | Stress | Full_Comp | 9 | 4.202444 | 3.163751 | CaHp Stress Full_comp | -2.24535 | 0.112159 |
| Weigelt et al. 2005 | *Carex arenaria* | Stress | Root_Comp | 9 | 3.110444 | 1.496866 | CaHp Stress Root_comp | -1.90024 | 0.091949 |
| Weigelt et al. 2005 | *Corynephorus canescens* | Ambient | Full_Comp | 8 | 19.49188 | 4.957775 | CcCa Ambient Full_comp | -0.21294 | 0.025419 |
| Weigelt et al. 2005 | *Corynephorus canescens* | Ambient | Root_Comp | 8 | 21.64729 | 2.616997 | CcCa Ambient Root_comp | -0.29824 | 0.009223 |
| Weigelt et al. 2005 | *Corynephorus canescens* | Stress | Full_Comp | 8 | 12.8665 | 5.064314 | CcCa Stress Full_comp | -0.12143 | 0.0332 |
| Weigelt et al. 2005 | *Corynephorus canescens* | Stress | Root_Comp | 8 | 12.09657 | 4.168549 | CcCa Stress Root_comp | 0.223325 | 0.028858 |
| Weigelt et al. 2005 | *Corynephorus canescens* | Ambient | Full_Comp | 8 | 19.49188 | 4.957775 | CcCc Ambient Full_comp_monoculture | -1.53153 | 0.046423 |
| Weigelt et al. 2005 | *Corynephorus canescens* | Ambient | Root_Comp | 8 | 21.64729 | 2.616997 | CcCc Ambient Root_comp_monoculture | -1.17259 | 0.005448 |
| Weigelt et al. 2005 | *Corynephorus canescens* | Stress | Full_Comp | 8 | 12.8665 | 5.064314 | CcCc Stress Full_comp_monoculture | -1.24792 | 0.0636 |
| Weigelt et al. 2005 | *Corynephorus canescens* | Stress | Root_Comp | 8 | 12.09657 | 4.168549 | CcCc Stress Root_comp_monoculture | -1.2265 | 0.086375 |
| Weigelt et al. 2005 | *Corynephorus canescens* | Ambient | Full_Comp | 8 | 19.49188 | 4.957775 | CcHp Ambient Full_comp | -0.34947 | 0.014878 |
| Weigelt et al. 2005 | *Corynephorus canescens* | Ambient | Root_Comp | 8 | 21.64729 | 2.616997 | CcHp Ambient Root_comp | -0.37287 | 0.012751 |
| Weigelt et al. 2005 | *Corynephorus canescens* | Stress | Full_Comp | 8 | 12.8665 | 5.064314 | CcHp Stress Full_comp | -0.79627 | 0.065046 |
| Weigelt et al. 2005 | *Corynephorus canescens* | Stress | Root_Comp | 8 | 12.09657 | 4.168549 | CcHp Stress Root_comp | -0.88276 | 0.054337 |
| Weigelt et al. 2005 | *Hieracium pilosella* | Ambient | Full_Comp | 8 | 6.659875 | 2.536238 | HpCa Ambient Full_comp | 0.278324 | 0.040514 |
| Weigelt et al. 2005 | *Hieracium pilosella* | Ambient | Root_Comp | 8 | 12.036 | 2.948714 | HpCa Ambient Root_comp | -0.36944 | 0.023997 |
| Weigelt et al. 2005 | *Hieracium pilosella* | Stress | Full_Comp | 8 | 9.403143 | 3.461359 | HpCa Stress Full_comp | -0.14418 | 0.038069 |
| Weigelt et al. 2005 | *Hieracium pilosella* | Stress | Root_Comp | 8 | 10.918 | 4.373947 | HpCa Stress Root_comp | -0.36312 | 0.042909 |
| Weigelt et al. 2005 | *Hieracium pilosella* | Ambient | Full_Comp | 8 | 6.659875 | 2.536238 | HpCc Ambient Full_comp | -1.54307 | 0.046112 |
| Weigelt et al. 2005 | *Hieracium pilosella* | Ambient | Root_Comp | 8 | 12.036 | 2.948714 | HpCc Ambient Root_comp | -1.99168 | 0.023622 |
| Weigelt et al. 2005 | *Hieracium pilosella* | Stress | Full_Comp | 8 | 9.403143 | 3.461359 | HpCc Stress Full_comp | -2.19488 | 0.046015 |
| Weigelt et al. 2005 | *Hieracium pilosella* | Stress | Root_Comp | 8 | 10.918 | 4.373947 | HpCc Stress Root_comp | -1.80304 | 0.093072 |
| Weigelt et al. 2005 | *Hieracium pilosella* | Ambient | Full_Comp | 8 | 6.659875 | 2.536238 | HpHp Ambient Full_comp_monoculture | -0.83619 | 0.050516 |
| Weigelt et al. 2005 | *Hieracium pilosella* | Ambient | Root_Comp | 8 | 12.036 | 2.948714 | HpHp Ambient Root_comp_monoculture | -1.86524 | 0.040972 |
| Weigelt et al. 2005 | *Hieracium pilosella* | Stress | Full_Comp | 8 | 9.403143 | 3.461359 | HpHp Stress Full_comp_monoculture | -1.60791 | 0.06047 |
| Weigelt et al. 2005 | *Hieracium pilosella* | Stress | Root_Comp | 8 | 10.918 | 4.373947 | HpHp Stress Root_comp_monoculture | -1.51224 | 0.091446 |
| Wilkinson & Gross 1964 | *Trifolium repens* | Ambient | Full_Comp | 12 | 7.29 | 1.877087 | Impute std | -0.07696 | 0.011969 |
| Wilkinson & Gross 1964 | *Trifolium repens* | Ambient | Full_Comp | 12 | 7.29 | 1.877087 | Impute std | -1.95694 | 0.282291 |
| Wilkinson & Gross 1964 | *Trifolium repens* | Ambient | Root_Comp | 12 | 7.29 | 1.877087 | Impute std | -0.64625 | 0.025647 |
| Wilkinson & Gross 1964 | *Trifolium repens* | Ambient | Shoot_Comp | 12 | 7.29 | 1.877087 | Impute std | -1.53543 | 0.124646 |
| Wilkinson & Gross 1964 | *Trifolium repens* | Stress | Full_Comp | 12 | 2.01 | 1.877087 | Impute std | 0 | 0.145353 |
| Wilkinson & Gross 1964 | *Trifolium repens* | Stress | Full_Comp | 12 | 2.01 | 1.877087 | Impute std | -2.30757 | 7.413209 |
| Wilkinson & Gross 1964 | *Trifolium repens* | Stress | Root_Comp | 12 | 2.01 | 1.877087 | Impute std | -0.88446 | 0.498894 |
| Wilkinson & Gross 1964 | *Trifolium repens* | Stress | Shoot_Comp | 12 | 2.01 | 1.877087 | Impute std | -1.39128 | 1.247162 |
