# Supplementary material for: Root and shoot competition lead to contrasting competitive outcomes under water stress: A systematic review and meta-analysis
Source: PLoS One. 2019 Dec 11;14(12):e0220674. doi: 10.1371/journal.pone.0220674 (PMC6905553; doi:10.1371/journal.pone.0220674)
Supplement: S3 Table — (DOCX) [file pone.0220674.s003.docx]

**SI Table 3. Koricheva & Gurevitch 2014 Meta-analysis checklist**

| **Recommended Item** | **Performed** |
| --- | --- |
| 1. Has formal meta-analysis been conducted (i.e. combination of effect sizes using standard meta-analytical methodology) or is it simply a vote count? | Meta-analysis performed with in the systematic review |
| 1. Are details of bibliographic search (electronic data bases used, keyword combinations, years) reported in sufficient detail to allow replication? | Yes, see materials and methods and PRISMA flow diagram |
| 1. Are criteria for study inclusion/exclusion explicitly listed? | Yes, see PRISMA diagram |
| 1. Have standard metrics of effect size been used or, if non-standard metrics have been employed, is the distribution of these parameters known and have the authors explained how they calculated variances for such metrics? | Yes, standard metrics – log response ratio (Hedges et al. 1999) |
| 1. If more than one estimate of effect size per study was included in the analysis, has potential non-independence of these estimates been taken into account? | Not accounted for |
| 1. Have effect sizes been weighted by study precision or has the rational for using unweighted approach been provided? | Variance of LRR is takes in account sample size and thus the precision |
| 1. Have statistical model for meta-analysis and the software used been described? | Yes, see materials and methods |
| 1. Has heterogeneity of effect sizes between studies been quantified? | Yes, see table 2 |
| 1. Have the causes of existent heterogeneity in effect sizes been explored by meta-regression? | Yes, see materials and methods |
| 1. If effects of multiple moderators have been tested, have potential non-independence of and interactions between moderators been taken into account? | Not accounted for |
| 1. If meta-analysis combined studies conducted on different species, has phylogenetic relatedness of species been taken into account? | Not accounted for given the 10 species |
| 1. Have tests for publication bias been conducted? | Yes: Rank Correlation Test for Funnel Plot Asymmetry using the “ranktest” function in the “metafor” package. We identified publication bias (Kendall's tau = 0.153, p = 0.045) |
| 1. If meta-analysis combines studies published over considerable time span, have possible temporal changes in effect size been tested? | There is a large time range (41 years), but this hasn’t been accounted for |
| 1. Have sensitivity analysis been performed to test the robustness of results? | Sensitivity analyses were not performed |
| 1. Have full bibliographic details of primary studies included in a meta-analysis been provided? | Yes, see PRISMA checklist |
| 1. Has the data set used for meta-analysis, including effect sizes and variances/sample sizes from individual primary studies and moderator variables, been provided as electronic appendix? | Yes, see SI Table |
