# Supplementary figures and images for: Root and shoot competition lead to contrasting competitive outcomes under water stress: A systematic review and meta-analysis
Source: PLoS One. 2019 Dec 11;14(12):e0220674. doi: 10.1371/journal.pone.0220674 (PMC6905553; doi:10.1371/journal.pone.0220674)

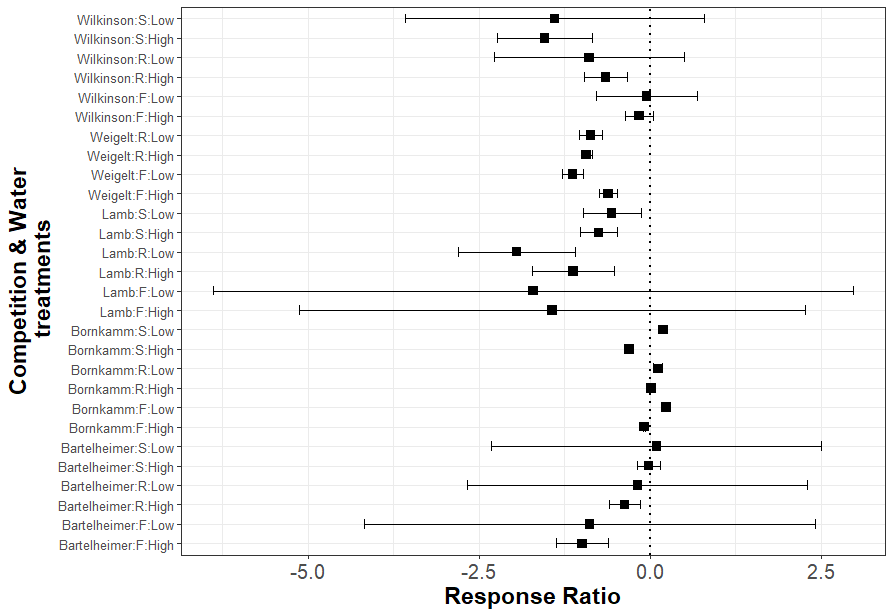

Supplement: S1 Fig — (TIFF) [file pone.0220674.s004.tiff]
